# Foreign Summary

**Published:** 1840-03-21

**Authors:** 


### FOREIGN SUMMARY.

*Report of the Proceedings of the Scientific Meeting held at Pisa in October*, 1839. By GEORGE STEWART NEWBIGGING, M.D., F.R.C.S.E., &c., in a Letter to the Editor.—Having been at Pisa during the late meeting of the Italian Scientific Association, I shall give you such short details of its proceedings as I think will interest you. A full account of its transactions will, I believe, be published by and by; but, as some time may elapse before that be ready for circulation, you may, perhaps, in the meantime, be pleased to have such notice of them as I, a bird of passage in Italy, have been able to glean.

The institution of this Association is, it is said, mainly to be attributed to the exertions of the Prince of Musignano, (son of Lucien Bonaparte, Prince of Canino,) who is devoted to the science of zoology, and reported to have a fine collection of natural history. He, it seems, attended the Freyburg meeting of 1838, and, judging from the good effects of such reunions in Germany, that a similar one in Italy might be beneficial to science, he set about its establishment, and was fortunately seconded in his views by the liberal encouragement which he met with on the part of the Grand Duke of Tuscany. It has, however, as was to be expected in this country, met with some opposition in certain quarters, but that has been of limited amount, and, with the exception of Modena, Naples, and the Papal dominions, the meeting was attended by professors, delegates, and others, from all the Italian states, making up the number of 421, foreigners and natives included. Of strangers, there were not many, and with the exception of Professor Oken, of Zurich, Professor Audouin, of Paris, and one or two more, none of any great celebrity attended from other countries, so that the meeting was essentially what, on our admission cards, it professed to be:—"La prima Riunione de Naturalisti, Medici, et Altri Scienyiati Italiani tenuta in Pisa nell' Ottobre, 1839,"—the first meeting of Naturalists, Physicians, and other scientific Italians, held in Pisa in October, 1839. The muster of these was numerous, among whom were many of at least an Italian reputation; but as the enumeration of them would be an unnecessarily tedious detail, we shall consign them to an honourable silence, except in so far as allusion is made to them in connection with the transactions of the meeting.

The first assembling of the members took place on the first of October, after having previously in a body, attended high mass, celebrated in the magnificent old Cathedral. This meeting was, of course, merely of a general and introductory nature, at which the general President and Secretary were named,—the former, Dr. Gerbi, Professor of Mechanical Philosophy, and the latter, Signor Filippo Corridi, Professor of Geometry and Trigonometry, both of the University of Pisa. Temporary presidents for each section were then appointed, under whose auspices these met next day, the 2d of October, in their respective localities, and proceeded to elect, by vote, the individuals who were to conduct their proceedings, during the session of the Association.

The following is a list of the sections into which the members divided themselves, with the names of the different Presidents and Secretaries:—

*Medicine.*

*President*—Giacomo Tommasini, Professor of Clinical Medicine at Parma.

*Secretary*—Francesco Puccinotti, Professor of Medical Jurisprudence at Pisa.

*Chemistry, Mechanics, and the Mathematical Sciences.*

*President*—Pietro Configliachi, Professor of Mechanical Philosophy at Pavia.

*Secretaries*—Luigi Pacinotti, Professor of Experimental Mechanics at Pisa, who took charge of the Chemical and Physical Departments of this Section.

Vincenzo Amici, Professor of Astronomy at Florence, who superintended the Mathematical and Astronomical subdivision.

*Agriculture and Tecnologia.*

*President*—The Marquis of Ridolfi,* having for

*Vice President*—Giuseppe Gazzeri, Professor of Chemistry at Florence.

*Secretary*—Francesco Gera, of Brescia.

*Zoology and Comparative Anatomy.*

*President*—The Prince of Musignano.

*Vice President*—Giacinto Carena, Secretary of the Royal Academy at Turin.

*Secretary*—Giuseppe Gene, Professor of Zoology at Turin.

*Botany and Vegetable Physiology.*

*President*—Gaetano Savi, Professor of Botany at Pisa.

*Vice President*—Giuseppe Moretti, Professor of Botany, and Rector of the University of Pavia.

*Secretaries*—Bartolommeo Biasoletto, of Dignano, superintending the Botanical subdivision.

Fillippo Narducci, Professor of Botany at Marerata, that of Vegetable Physiology.

*Geology, Mineralogy, and Geography.*

*President*—Angelo Sismondi, Professor of Mineralogy at Turin.

*Secretary*—Ludovico Pasini, of Vicenza.

The election of the preceding presidents constituted the sectional proceedings of the second day; after which, as a kind of gala, the inauguration took place of a statue† of Galileo, lately put up in the court of the University. This ceremony consisted in a chorus, singing several songs composed for the occasion, and in the delivery of an appropriate and very eloquent oration by Signor Giovanni Rosina;‡ the intervals being beguiled by two

* The Marquis of Ridolfi is well known in Italy as the possessor of the property of the Meleto, situated between Florence and Siena, where he has established a school for Agriculture, and where are held the annual meetings of the Tuscan Agricultural Society.

† By Derni, an artist of Florence.

‡ Author of various literary works, among which are some comedies; the historical novels of Luisa Strozza and La Monaca di Monya; and a history of painting in Italy, in which he is now engaged.

bands of music, which from time to time relieved each other. As the day was one of Italy's own, bright and smiling, and the attendance numerous, with a reasonable sprinkling of the fair sex, the whole was a very gay and interesting scene.

This summed up the doings of the second day.

On the third there was a general meeting, at which, besides the members themselves, many ladies and strangers were present, whom curiosity or love of science had brought together. At this the general president read his address; and it was not till the fourth day of the Congress that its real business, so to speak, commenced in its different sections.

The first meeting of the medical section, of whose proceedings I propose giving you a few particulars, was opened by an address from its President, Professor Tommasini, in which, among other topics, he set forth the advantages of correct observation and strict deduction in the science of medicine, and enlarging on the benefit of such assemblies as the present among medical men, concluded by expressing his best wishes for the progress of science, and for the future continuance and welfare of such associations.

It is unnecessary to detail the transactions of this section in chronological order from day to day, and I therefore adopt the more simple arrangement of dividing into two categories the most important communications made at the different meetings, noticing shortly as I proceed any interesting discussion to which they gave rise. These may accordingly be divided into, 1. Those relating to personal observations or experiments; and, 2. Memoirs read to the section.

And now for the first class, namely, Personal Observations or Experiments.

*Dr. Corneliani*, Professor of Clinical Medicine at Pavia, and *Dr. Polli*, Chemist of Milan, read papers on Diabetes, the one giving his observations on the history and nature of that disease, while the other detailed the result of his analysis of the chyme formed in the stomach of diabetic patients.

Dr. Corneliani's views may thus be briefly summed up. He considers that the essential conditions of diabetes consist in an abnormal state of the stomach, during which sugar is eliminated in this viscus and not in the kidneys; that the use of creosote is highly useful in this affection, in order to remove the deranged condition of innervation of the stomach, to fix the albumen in the blood, and prevent the failure of nutrition; that the cotemporaneous use of an animal diet is also necessary, in order to restore the homogeneous materials to the peculiar assimilating power of the diseased organ.

Dr. Polli viewed diabetes as in no case having its seat in the kidneys, but as being always an affection of the stomach. This, according to him, consists not in inflammation, nor in irritation, nor in weakness of that organ, but is a deviation from its physiological state, which may be more properly regarded as a gastric neurosis of some specific character. He was of opinion, that where inflammation, congestion, or other such conditions of the stomach present themselves in the course of this disease, they ought to be viewed as its consequences or accidental concomitants. He thus concluded, from numerous analyses which he has made of the chyme, that diabetes is a specific affection of the stomach, which he calls an abnormal process of saccharification.

*Dr. Taddei*, Professor of Pharmacology at Florence, read a memoir on the colouring matter, or Hæmatosine, of the Blood. After alluding to the uncertainty which even at this day prevailed in regard to much of our chemical knowledge of this fluid, he adverted to the want of accurate information regarding the true nature of hæmatosine,—some of the best organic chemists, and Berzelius among the rest, having gone the length of asserting, that if hæmatosine could be at all distinguished from albumen, it was so only by its greater coagulability; asserting at the same time, (having regard to the manner in which they were both acted on by chemical reagents,) that the one was a mere modification of the other. Professor Taddei considered the hæmatosine or colouring matter to be essentially different from the albumen, and if down to the present day they have appeared similar, it is because the former has never been obtained pure, but has had its properties always concealed or masked by the albumen with which it is constantly found combined.

Though it be difficult to obtain it pure, yet Dr. Taddei conceives he has discovered a method by which the hæmatosine may be procured free from any admixture. This process he calls that of Interposition, (Interposizione,) because in making use of a strong acid, as the sulphuric, he moderates its too energetic action by interposing various substances; first carbonate of soda, and then sulphate of copper, among the molecules of the colouring matter, and those of the albumen, and thus succeeds in so separating these as to be able to dissolve the hæmatosine with alcohol, and leave the albumen in the form of a matter presenting the appearance of the soft of bread.

Hæmatosine thus procured, presents very different characters from those which have been usually assigned to it. It has been generally pronounced coagulable by heat, and soluble by water;* but, on the contrary, Dr. Taddei finds the substance procured by his process to be *not* coagulable by heat, nor acids, nor alcohol. It is insoluble in water, but soluble in alcohol, and in ether, and, above all, in alcohol

* Despretz, Engelhart, Thenard, Guy-Lussac, Berzelius.

acidulated by nitric acid ; it is also soluble in alkalis, in which it becomes of a deep-green colour, not unlike that of the bile. It always appears red when seen by a refracted light. It combines readily with various salts, and is precipitated with them from the different solutions ; it has a strong affinity for albumen and other coagulable matters, from which it is almost inseparable after combination. It is deprived of colour by carbon ; it shows the iron, which it contains in abundance, after having been treated by chlorine ; but if it be dissolved by potassa or soda, the chlorine no longer succeeds in showing or rendering the iron soluble, that being retained by the same hæmatosine which is now precipitated white instead of red.

The pure hæmatosine has a colouring property of considerable strength, and is in very small proportion when compared with the other elements of the blood.

Dr. Taddei added, that he first made known his process in 1836, in his public course of pharmacology at Florence. He is aware that Mons. Lecanu presented a thesis to the Faculty of Medicine of Paris in the end of 1837, where he showed a new process of obtaining the colouring matter of the blood, which is not, however, based on *Interposition*, although sulphuric acid be employed. The hæmatosine of Lecanu, moreover, did not present all the properties of that which Dr. Taddei procured by his process.

*Dr. Federici* of Messini presented to the section some observations on the nature and causes of Dry Gangrene, in which he advanced the hypothesis of its proximate cause being an antiperistaltic movement of the capillary arteries, founding his views on some experiments which he had made on worms and frogs, in which the administration of spurred rye had produced a retrograde motion of the blood.

*Dr. Linoli* of Tuscany read a paper, in which he attempted to prove that inflammation has nothing to do in the reproduction of organized parts, illustrating his arguments by observations made on the union of bone after fractures. He thought that all depends on the transudation of fibrine from the capillaries, and its subsequent consolidation ; that there is never a new product formed ; but that all takes place at the expense of the fractured bones, during which they themselves are always reduced in bulk near the point of fracture.

On this question, Dr. Betti, surgeon to the Hospital at Florence, was of a different opinion. He could prove by anatomical preparations in the Florentine Museum, that there was a true reproduction of bone in the case of the extremities, where the fractured parts were in apposition, but he thought that there was no reproduction in those cases of fracture of the cranium, where there was much loss of substance. Dr. Corneliani of Pavia maintained with less limitation than Dr. Betti, that reproduction of bone takes place in fracture of bones of the cranium, basing his views on pathological specimens to be seen in the Museum of Pavia.

*Dr. Giulj*, Professor of Natural History and Botany at Siena, and Physician at the Baths of Monte Cateni, (not far from Florence,) communicated the result of a series of observations which he had made on the electrical state of different organs of individuals employing these* mineral waters. He has found on applying the electrometer over the region of the liver, for example, that it has indicated a state of electricity after the use of the waters, different from what it did before their employment.

This notice gave rise to some remarks from Professor Puccinotti, who entered at some length, into the consideration of the actual state of science in regard to the application of electricity to physiology and pathology. He endeavoured to show that all the observations hitherto made were deficient in one very important point, namely, the demonstration of an electrical current in the nerves and muscles of hot blooded animals. With regard to the observations of Dr. Giulj, they did not appear to prove any thing at all, because the application of silver plates on the different regions of the body, in connection with the conductors of the galvanometer, could only tend to show the state of the skin's electricity, and could in no way indicate that of an organ within the chest or abdomen. In conclusion, he expressed his conviction that the chemical and physical sciences were now a-days in a better condition than at any former period, for furnishing useful lights to the science of medicine ; but that he who would employ them must know how to avail himself of these auxiliary sciences, being duly acquainted with what they are capable of doing, and not claiming for them powers, their possession of which is not yet established.

*Dr. Comandoli* made some remarks on affections almost all of an inflammatory character, which were in his opinion calculated to show the importance of the principles on which is founded the system of Italian pathology.

*Dr. Pacini* of Pistoja showed on a dead body the existence of certain small ovular bodies attached to the subcutaneous branches of the nerves of the palm of the hand, of which he considered himself the discoverer, and which he thought are probably connected with the sense of touch.

*Dr. Schinas*, Professor of Clinical Medicine at Malta, read a memoir on Dysentery, with observations on the specific action of calomel on that affection, given in large doses of half a drachm.

Some remarks were made on this subject by Professor Tommasini and Dr. Del Chiappa,

* The salts with which they are impregnated, besides iron, are chiefly muriate of soda, carbonate of magnesia, and lime.

Professor of Clinical Medicine at Pavia, both of whom brought forward cases illustrative of the good effects of such a mode of treating dysentery.

*Dr. Schinas* also made some remarks on Tetanus, whose pathology he considers to be an inflammation of the spinal marrow,—a view which he has been led to take from the observation of twelve cases. In support of his opinion, he showed some drawings to the meeting, illustrating the engorgement of the vessels of the spinal marrow, as taken from patients who had died of tetanus.

*Dr. Pecchioli*, Professor of Clinical Surgery at Siena, gave some statistics in regard to the cases of stone in the bladder, which have occurred in his practice at the hospital of that town.* He has had 72 cases, 11 of which, not operated upon, were dismissed relieved; 2 of them (women) having passed the calculi spontaneously. Of the 61 remaining, 58 were men, and 3 women; and of these 47 were operated upon by cystotomy, and 14 by lithotrity. His proportion of deaths has been somewhat less than six per cent.

| *Electro-vital Current.* | *Current obtained from parts possessing Different Chemical Properties.* | *Current obtained after Death from Different Organized Parts.* |
|---|---|---|
| 1. Cannot be obtained by sinking wires into the animal experimented on, nor by the simple application of plates of metal to its organs. | 1. Is obtained by simple contact, on placing plates on the membranes, or the surface of the organs. | 1. Can be obtained, but not constantly, and always on applying the conductors in any way to different parts of the body. |
| 2. Is strong, and manifests itself promptly, causing at the same time a violent reaction in the living animal. | 2. The manifestation of the current is not accompanied by any reaction on the part of the animal during life or after death. | 2. If the parts be not moistened by some decidedly alkaline or acid matter, the current is at first scarcely appreciable, sometimes not detected at all, and sometimes of a strength indicated by not more than two or three degrees of the galvanometer. |
| 3. Is materially weakened if the anatomical proceedings be not adroitly performed, or the animal lose much blood in the course of them. | 3. Is not influenced or weakened by the anatomical proceedings, as may be seen in the experiment of forming the communication between the stomach and gall-bladder during life. | 3. Is somewhat increased by bringing a larger portion of the blades in contact with the parts of the animal to which they are applied. |
| 4. It has an energy bearing some relation to the struggles of the animal. | 4. Its energy is always the same, bearing no relation to the struggles made by the animal. | 4. It is also increased by lessening the space which intervenes between the points of insertion of the two blades. |
| 5. According as the instantaneous struggle of the animal is stronger, so is the great energy of the current on the first plunging of the lancets. | 5. Its energy is nearly the same, whether the first time that contact is made, or in succeeding trials. | 5. If the intervening space between the conductors be increased, the current becomes weaker, and at last dies away entirely. |
| 6. Its energy decreases and becomes extinct, as nervo-muscular life fades and dies. | 6. It appears to have no dependence on the vigour of life, but may be rendered weaker by wiping or washing the surface (*e. g.* the mucous membrane of the stomach) to which the blades are applied. | 6. The current becomes stronger as the *post mortem* changes progress, but is always notably weaker, even at its maximum, before putrefaction, than the other two currents. |
| 7. The current is always in the same direction. | 7. Its direction is variable, according to the different electrical states of the parts to which the conductors are applied, and if the relative position of these be changed, the current is also inverted. | 7. The direction of the current is variable, and inverted as the conductors are inverted. |

* This table was not very minutely drawn up, and afforded no results of any importance.

Some experiments* were shown at a meeting of this section by Professors Puccinotti and Pacinotti, which were also repeated at the section of Chemistry and Physics, tending to show the existence of an electro-vital current in hot-blooded animals, Professor Puccinotti having found a mode of deriving it from the nervo-muscular system. By this method he has rendered it appreciable by the electrometer of Nobili to the extent of 60°, and upon one occasion as high as 90° of that of Melloni. His manner of proceeding consists in simultaneously inserting the lancet-shaped blades of platinum (which communicate with the galvanometer in the usual way,) one into the brain, and the other into a muscle of the extremities of the animal on which he experiments, as, for example, a cat, a rabit, or a pigeon. By means of this instantaneous plunging of both lancets into an animal whose physiological condition is sound, a strong reaction takes place, during which the current leaves its natural conductors, and attaches itself to the metallic ones. This current presents characters analogous to the physiological changes of the animal itself, and may be distinguished from other currents demonstrable in the subject of experiment, with which it might at first view be considered identical. Thus, Professor Puccinotti demonstrates what he considers an electro-chemical current by cutting open the abdomen of the animal during life, when, having exposed the cavity of the stomach, he applies one of the plantinum blades to its mucous membrane, while, at the same time raising the liver against the diaphragm, he applies the other blade to the gall-bladder, calculating that he has thus obtained two surfaces in different states of electricity; the stomach more or less bedewed with gastric juice, being acid, while the gall-bladder is more or less alkaline. In this way a current of electricity is manifested by the galvanometer, which is stronger than the electro-vital, and appears to obey different laws. He also obtains another current after the death of the animal, which he considers different from the two preceding. Immediately after decapitation he introduces one blade into the neck, and the other at the same instant into one of the extremities of the animal experimented on, when a current of electricity is detected, which is much weaker than either of the preceding, and is apparently subservient to different laws. To show in what consist these differences, according to Dr. Puccinotti, the tabular view of them in the preceding page is given nearly in the Professor's own words.

In this first category of observations, should be mentioned the memoirs laid before the section relative to orthopedy.

*Dr. Pravay* of Lyons, an individual who cultivates this branch of surgery with great zeal and reported success, brought forward his views in regard to the curability of congenital luxations of the head of the femur, which had long been considered in France as incapable of effectual permanent reduction. He considered such deformities as within the reach of surgery, but that patience and time is necessary in order to secure success, of which he gave an example in a case where he had effected permanent reduction, after a duration of treatment to the extent of seven months. At first, the case was most discouraging and obstinate, as the head of the femur, for several days consecutively, jerked upwards and backwards on the dorsum of the ilium every time it was brought down to its normal position.

*Dr. Scalvanti* of Pisa read an account of some successful cases of cure of club-foot, showing at the same time the apparatus he had employed, and in some cases without section of the tendons.

A mummy was shown to the section by *Dr. Pacini* of Lucca, which was in a state of good preservation, though prepared five years ago. The mode he adopted was that of Tranchina of Sicily.

*Dr. Comi* of Rome showed some preparations of animal matter which he had reduced to a state of stony solidification, preserving at the same time the original form of the parts so prepared. He supposed his process to be the same as that adopted by Segata.

The second class of the proceedings of this section, according to the arrangement adopted, was composed of written dissertations.

*Dr. Giacomini*, Professor of Clinical Medicine at Padua, read a paper on the nature and vitality of the Human Blood, in which, after having commented on the uncertainty of all chemical and physical examinations of its nature, he concluded that it is a homogeneous fluid, and that its vitality depends upon the solids. He thought also that its alterations are never spontaneous or primary, but are merely symptoms, the production of which must be looked for in the primary alterations of the solids.

*Dr. Bufalini*, Professor of Clinical Medicine at Florence, in reference to this paper, observed, that whatever might be the unsatisfactory character of the chemical and physical researches into the nature of the blood, they by no means excluded the possibility of finding, by their aid, some principle constituting the primary alteration of this fluid, and being thus the spontaneous cause of disease—that the facility with which the blood becomes altered in its properties is itself a proof of the susceptibility of this fluid to morbific causes. Professor Bufalini thought there could be no doubt that some diseases are of humoral origin—that the animal economy ought to be regarded as subject to the influence of dynamic and chemical actions—and that the primary seat of a disease should not be sought by having recourse

* A report of these was directed to be printed.

to the principle of vitality alone, but also in the analogies which the chemical and physical sciences furnish to medicine.

*Dr. Turchetti* read some considerations on the bad moral condition of the Medici di Condotta of Italy.

These are a class of medical men, who are appointed and paid by the village or community in which they practise, receiving from it an annual income for their trouble, but not entitled to demand remuneration from individuals.

*Dr. Morelli*, Professor of Clinical Medicine at Pisa, read some remarks on the value of the physiological works of Professor Forni of Turin, which he thought were not so extensively known or so highly appreciated as they deserved to be.

*Dr. Ferrario*, of Milan, read a paper on the utility and necessity for the interests of medical science of some arrangement in every hospital, whereby the value of the different pathological systems might be fairly tested, and for this purpose suggested that a statistical report might be made, containing the respective pathological, therapeutic, and clinical observations made in each ward. For example, a clinical ward might be allotted in every hospital for the patients of those individuals upholding inflammation as the cause of all disease, another for the "Mistionists;" and so on, with regard to every system worthy of investigation—that of homœopathy being excluded by the undivided verdict of the meeting. Dr. Ferrario showed a programme of such a statistical table as he suggested might be adopted in all the hospitals of Italy.

This communication was followed by some observations of pathological and clinical interest, as to the construction of the most useful form of a table of medical statistics for Italian hospitals, in which Drs. Bufalini and Ferrario took part.

*Dr. Fassetta*, Physician to the Civil Hospital of Venice, brought forward some observations on the combination of the moral with the medical treatment of insane patients; presenting at the same time a statistical account of the doings of the female lunatic asylum at Venice, during the years 1837 and 1838, regarded under the influence of moral causes.

*Dr. Bouros*, Professor of Pathology and Clinical Medicine at Athens, gave the results of of his chemical analysis of the principal hot springs of Greece, with some account of their medical uses, aud geographical and geological notices of their localities.

*Dr. Meneghini*, Assistant to the Botanical chair at Padua, read an article on Phrenology, in which he expressed his conviction that the reason of that doctrine suffering in the opinion of some learned and impartial men, is traceable to the circumstance of many phrenologists, since the time of Gall, forgetting the fundamental precept of their master, to take anatomical facts for their guide, and to weigh well the deductions drawn from cranioscopy. Having taken a short and rapid view of the structure of the brain, he showed, with the aid of comparative tables, that the prominences of various regions of the cranium, instead of being produced by the convolutions immediately under them, might often owe their origin to some extraordinary development of deep-seated parts.

The discussion at the last meeting of this section, 14th October, was sustained mainly by Professor Puccinotti and Dr. Comandoli, who endeavoured to combine all the different theoretical propositions advanced during the previous sederunts. Professor Puccinotti attempted to show that all such had the tendency to give a character of general agreement to certain cardinal points on which the Italian pathology is founded, and that those individuals did not properly understand it who considered this system as divided by doctrines fundamentally different or opposite. On such a harmony of principles he conceived depended the expectations and hopes of the ultimate utility and excellence of such reunion as the present.

Besides these proceedings there were others which do not come under either of the above categories, but these were few or of little consequence. Among the first were some prizes offered by different individuals of the Section.

Joseph Frank* proposed a prize of 500 francs for the best essay on the Hippocratic system of medicine, to be decided at the next meeting of the association.

Dr. Thaon of Leghorn also proposed one to be awarded to the individual who would bring forward the greater number of satisfactory observations on a method of curing carcinoma of the breast.

Thus terminated the proceedings of the medical section of this association, of which the above is a somewhat curtailed account. Their meetings were interrupted, as well as those of all the other sections, on the eighth day of the Congress, by a general meeting similar to the first, at which different letters were read, and the names mentioned, of various individuals who had come to the meeting of the association as deputies from different academies or universities. These were in all upwards of forty, composing sixteeen deputations. The Rev. Raffaello Lambruschini then read a paper on the part which the Earth acted in preparing and supplying the Nutrient Juices to Plants. He was followed by the Prince of Musignano on the Electrical Properties of the Torpedo,—by Professor Belli of Milan, on the Formation

* Son of Peter Frank, who was long connected with the schools of medicine of Pavia and Vienna, as was also Joseph himself, though subsequently Professor of Practical Medicine in the University of Wilna. He has now for many years abandoned his public pursuits and retired for the most part to a property he has on the banks of the Lake of Como.

of Hail,—by Professor Domnandos, deputy from the University of Athens, who brought forward some geological observations on the Island of Santorino,—and lastly, Tommasini read a memoir on the Influence of Habit on the Animal Economy, both in its Physiological and Pathological condition.

On the last day, (15th October,) another general meeting was held, at which the grand Duke of Tuscany, (who also attended one of the meetings of each of the sections,) attended, when the general and sectional secretaries read their reports, and some business of a miscellaneous character was gone through. The association having been announced to meet next year (1840,) at Turin, and the year following (1841,) at Florence, then closed its business for this session.

I have only to add, that all the arrangements throughout were distinguished by a regularity and fitness scarcely to have been expected in so early a stage of the association's existence, and that its members had great reason to be highly gratified by the steps taken for their accommodation and amusement during their sojourn in Pisa. Every public place was thrown open for their inspection, and they had free ingress to the Cassino of the *good* town of Pisa, where newspapers and journals were found all day, and in the evening, dancing or conversation reunions. They were likewise honoured by an invitation to dine in the Duke's Palace, with the Governor of the city, of which the greater part availed themselves, and drank most enthusiastically to the owner's health.

Each member on reclaiming his passport which he had deposited on enrolling his name, also received as a souvenir of this first establishment of the association, a medal, along with a printed copy of Rosini's address, and other pamphlets connected with the proceedings of the Congress. The medal is stamped on one side with the head of Galileo, and on the other, with the architectural group for which Pisa is so justly famed, namely, the Cathedral, Campo Santo, Campanile, and Baptistery,—above which is the inscription: A onore di Galileo, Pisa,—memore del primo consessu dei Naturalisti Italiani auspice Leopoldo II, Ottobre, 1839.—*Ed. Med. and Surg. Journal.*

---

*The Anasarca which follows Scarlatina.*—At the Westminister Medical Society, Mr. Snow read a paper on the anasarca which follows scarlatina. He commenced by saying that there appeared to be great variety in the frequency and severity with which this affection followed scarlet fever, at different times, and in different places. During the late epidemic in the metropolis, the subsequent anasarca had been rather frequent, and somewhat fatal. Mr. Cruickshank first discovered albumen, or serum, in the urine of dropsical patients. Dr. Wells soon afterwards confirmed the fact, and made some progress in ascertaining the circumstances under which it was found. He stated his belief in the injurious effects of mercury in this class of dropsies, and showed that the administration of that medicine would often cause albumen to be secreted with the urine; and he also ascertained that in every, or almost every, case of anasarca following scarlet fever, the urine contained serum, and generally the colouring matter of the blood also. Dr. Blackall had since extended this subject, and spoken particularly of the injurious effects of calomel in the anasarca which followed scarlet fever. More recently, Dr. Bright had discovered the connection between the albuminous state of the urine and certain diseases of the kidney, and had shown that such state of the urine was by no means confined to dropsical patients; but that dropsy was only one, out of several, secondary affections which might be occasioned by renal disease. Many physicians hesitated, at first, to accept these views, but they now, for the most part, admitted that, with a few recognisable exceptions, albumen in the urine indicated a granular or flaky deposit in the kidney; or a state of disease which, if not cured, might lead to this deposit. Dr. Bright and Dr. Christison both said that this disorganization of the kidney might be occasioned by scarlet fever, and one of them had related a well-marked case which had clearly dated its origin from that disease.

The author of the paper then related twelve cases which occurred during the last autumn. The first of these was one following a very severe form of scarlet fever, in a girl twelve years of age. It proved fatal in a very sudden manner, probably from extensive hydrops pericardii, which was found after death. There were also hydrothorax, ascites, and general œdema, with evidence of pleuritis in the presence of recently effused lymph. The contents of the cranium were healthy; the kidneys were so much hypertrophied, that they weighed a pound, avoirdupois; the left weighing eight ounces two drachms, the right, seven ounces six drachms. These kidneys were laid before the Society; one of them had been injected by Mr. Toynbee; the injection returned very readily by the veins, and portions of the organ, examined under the microscope, showed the Malphigian bodies to be very much enlarged, and the vessels composing them opened out, so that they no longer presented a properly-defined form.

The subject of the second case was the brother of the above patient, and was nine years of age; he had extensive anasarca and ascites, and recovered under a venesection to four ounces, and a course of free purgation. This boy had, during his illness, a peculiar dingy colour of the skin, which symptom also accompanied one of the other cases. This was occasionally a symptom of an advanced state of granular disease of the kidney, but Mr. Snow believed had not been previously noticed in an early stage of the affection.

Four out of the remaining cases proved fatal. In two of them an examination was made, and the kidneys were found to be much congested, though not hypertrophied or otherwise diseased; the mucous lining of the calices and infundibula, however, was in one of the cases much injected, as if from inflammation. In this case the lungs were also œdematous, and a portion of one lung hepatised, and there was evidence of pericarditis in lymph thrown out on the surface of the heart. One of the other cases, in which no autopsy had been made, appeared to terminate by œdema of the lungs.

The eleventh case detailed, was that of a delicate girl, eleven years of age, who was attacked with pain in the region of the heart, three weeks after scarlet fever; the heart pulsated with great force, one hundred and seventy times in the minute, and the pulse at the wrist was scarcely perceptible. The dulness, on percussion of the cardiac region, was much extended, probably from hydrops pericardii; there was a very slight œdema at the upper part of each foot, but no where else; the urine was scanty, opaque, and albuminous. Under the use of small doses of antimony and digitalis, with purgatives and counter-irritation of the chest, this patient gradually recovered.

The twelfth case was that of a boy, aged six years. About three weeks after a very slight attack of scarlet fever he was seized with difficulty of breathing and slight feverishness, having no pain or râle; two days afterwards he was attacked with spasmodic pain in the bowels and nettle-rash. The following day, the fourth of his illness, he had constant pain in the abdomen, with great tenderness on presure; his bowels were obstinately confined; countenance anxious; tongue dry and furred, and pulse frequent. Blood was drawn freely by leeches applied to the abdomen, and on the following day these symptoms were relieved, but the difficult breathing remained, and there was now pain in the cardiac region, and the heart was beating with great force and frequency. There was no œdema during any part of the illness; but considering there might be affection of the kidney, unaccompanied by the anasarca, the urine, which was secreted in diminished quantity, was examined and found to be albuminous. The urine soon regained its healthy state; but the pain and increased action of the heart continued, and a loud bellows sound became established with the first sound; the symptoms afterwards became somewhat relieved, but the boy was still under treatment.

Mr. Snow said that, in all these cases but two, albumen was found in the urine, and he believed it would have been detected in these also, at some period of the affection, if the urine had been examined more than once. The greatest quantity of albumen had been found in the most severe cases, and, with the commencement of recovery, it became less. In more than half the cases there was also more or less of the colouring matter of the blood. In renal affections, with albuminous urine, not following scarlet fever, the colouring matter of the blood was by no means so often present. He thought that this difference depended on the circumstance, that the affection, following scarlet fever, was always observed in its recent form, or else in the fact that it occurred chiefly amongst children. He had observed something which gave an opacity to the urine in almost every case, and which, in the course of a few hours, settled down in the form of a white flocculent deposit; this answered to the tests of fibrine, and he believed that it was the fibrine of the blood. In all the cases in which the specific gravity of the urine was ascertained, it was much below the standard of health; denoting a great deficiency in the quantity of urea, but the urine was, at the same time, very scanty; the deficiency of the urea must have been, therefore, very great; and it must have, consequently, accumulated in the blood, and it had often been detected there. He thought that this urea, either by direct contact, or through the influence of the nervous system, stimulated the heart to undue action, which would increase, if it might not cause the pleuritis, and the other inflammations so common in this affection; and if the increased action continued, it might lead to disease of the heart itself, or of the pericardium. In at least half the cases he had witnessed, he had found the action of the heart increased much beyond what sympathy, with disease in other organs, would account for; this increased action generally came on some time after the anasarca. There was pain in the region of the kidneys in only a few of the cases; in a few more there was tenderness on pressure; but in the majority there was neither pain nor tenderness. Vomiting occurred in many of the cases, and in several more there was another form of gastric irritation, viz., craving appetite. The tongue was clean in most of the patients. Pain in the belly had been a common attendant, even when the peritoneum was not distended with fluid.

Two of the cases occurred in a brother and sister, and a third patient had a sister who had been affected with the disease three years ago, facts, confirming the observation of Dr. Wells, that some families seemed to be predisposed to it more than others. He, perhaps, could offer no explanation why scarlatina predisposed to the affection; but as exposure to cold often seemed to be the immediate cause of the attack, it would be a prudent measure to defend the patients from cold as much as possible for a month, at the end of which time they might be considered out of danger. The majority of cases commenced about three weeks after the beginning of the fever, but several of them much earlier, and a few later. It seemed to follow mild as well as severe cases, without showing a preference to either. He recommended blood-letting in the commencement of the disorder, when the strength would bear it, and he alluded to several authorities in proof of the success of this practice. He advised, also, that free purgation should be resorted to in all cases, and gave the preference to the compound jalap powder as a purgative, and thought these measures would be assisted by the administration of digitalis.

Dr. Addison would offer one remark on the subject which had occupied the Society, and that was with reference to the assumption, that the kidney was the *fons et origo* of all the mischief in the cases (and those analogous to them) which had been read. The fact which Dr. Bright had established of the connection which existed between the mottled kidney and albuminous urine, with dropsy, was a very valuable one. He (Dr. Addison) thought, however, that as we gained experience, the sweeping conclusion, that the disease of the kidney was the *fons et origo* of the dropsy and other symptoms, would be found to be incorrect, and that we were, in fact, mistaking an effect for a cause. Mr. Snow, in his paper, has detailed one case in which the anasarca, and the albuminous state of the urine occurred simultaneously, showing that one was not dependent upon the other. Other cases also led to this inference. Again, cases occurred—he referred to those of a chronic kind—which presented a series of phenomena characteristic of disease of the kidney, but which were unaccompanied with the evidence of albuminous urine or of dropsy, yet the patient dying, Bright's kidney was found. He did not deny that the disease of the kidney produced the albuminous urine; but, in common with many other physicians, he doubted if that diseased state was the origin of dropsy, and the other series of phenomena which accompanied it. He should rather ascribe all these, including the disease of the kidney itself, to a peculiar state of the system, which might be induced by intemperance, and a variety of causes. When cases occurred in which the symptoms indicated the presence of a mottled kidney, it was remarkable how quickly the disorganization was detected; on the contrary, however, when there were no symptoms of such disease, the kidney was said to be sound, when a single glance would be sufficient to decide that the kidney really was in a mottled state.—*Lancet.*

---

*Prussic Acid in Phthisis.*—The effects of medicinal substances have so seldom been observed with exactitude that we are glad to record the following, from a recent number of the "French Medical Gazette."

M. Andral has employed prussic acid in various stages of pulmonary consumption. We select twenty-four cases, of different ages and sexes: fourteen of these were males, ten females. Of the former, one was 26 years of age; two, 23; one, 24; two, 27; one, 30; two, 34; one, 36; one, 39; one 45; and one, 52. The ages of the females, were 19, 27, 28, 33, 34, 36, 39, 48, and 64.

*Stage of the disease.*—*Males.* Three were affected with tubercles, in the first stage; in two, the tubercles were beginning to soften; in one, they had passed to the second stage; in two, small caverns had formed; and six were in the last stage of tubercular consumption.

*Females.*—In one, the tubercles were in the first stage; in two, in the second; and in seven, in the third stage.

*Dose.**—For the males, the lowest dose at which it was necessary to stop, was ten drops; one patient was able to take twenty-four drops; the others, from sixteen to twenty-four. When carried to the dose of twenty-eight drops, nervous accidents were produced. For females, the minimum dose was eight drops, the maximum, eighteen.

*Effects.*—In the greater number of cases, the effects of the remedy were absolutely null, and in some, the symptoms were even aggravated. Thus, in one female the vomiting was increased; in a male, the diarrhœa and colliquitive sweats became more frequent. In the female, aged 64, the abdominal pain was greatly aggravated. In some cases, each spoonful of the mixture occasioned severe coughing, and in others, dyspnœa.

Three patients were apparently relieved; the following is a brief account of their cases:—

*Case* 1.—A man, 52 years of age, affected with tubercles in the second stage, took the prussic acid for two months, the dose being gradually carried to 18 drops. He experienced some sensation of heat about the epigastrium, and slight feebleness. He affirmed that his cough was better, and felt so well that he left the hospital. There were, however, no signs of amelioration, so far as the symptoms were concerned.

*Case* 2.—A girl, 19 years of age, had tubercles in the first stage, but on the point of softening. The febrile symptoms were high; pulse one hundered and twenty; but the cough and dyspnœa were moderate. The dose was gradually carried to 16 drops, and continued, in this quantity, for a month. The medicine occasioned heat of surface, giddiness, and then sweating. The febrile symptoms gradually abated, and she left the hospital, certainly somewhat better, but with night sweats. M. Andral attributed her improvement, not to the medicine, but to the fact, that the softening of

* The acid employed was that prepared after the formula of Gea-Pessina. It contains one atom of anhydrous acid to five of water.

the tubercles was now completed, for the existence of small tubercular cavities was manifest.

*Case* 3.—A young man, 20 years of age, was admitted into La Charité, with tubercles in their first stage. He left the hospital, improved, in a few days, but the amelioration might as well have been attributed to rest and diet, as he had just returned, much fatigued, from a long voyage.

Fourteen of the patients died under treatment; seven were dismissed without having experienced any benefit. Hence, we may fairly conclude, that in the 24 cases just mentioned, the prussic acid exercised no influence over the symptoms or progress of pulmonary consumption.—*Lancet, from Gaz. Med. de Paris.*

---

*Greater number of Still-born in Illegitimate than in Legitimate Births.*—It is well known that unmarried females who become pregnant are much more likely to have still-born children than married women. Professor Jorg in his recent work, "Die Zurechnungs fähigkeit der Schwangern und Gebärenden beleuchlet," states that in Leipsic in 1835, there were born 1121 legitimate and 249 illegitimate children; and of these 45 of the former, and 28 of the latter were still-born; the ratio being in legitimate births, 1 still-born in nearly every 22 births, whilst in the illegitimate it was one still-born for a fraction more than every 13 births.—*Edinburgh Med. and Surg Journal.*

---

*Conception after cessation of the Menses.*—The subjoined case may be interesting in a medico-legal point of view, and tend in some measure to subvert the generally received opinion, that conception will not take place after the cessation of the catamenia.

Mrs. W., a healthy woman, aged 44, had, previous to her present pregnancy, given birth to nine children at intervals of from eighteen months to two years. She was confined with her ninth child in September, 1836. The secretion of milk had always before been plentiful, but on this occasion it was much diminished in quantity, and, in fact, the child weaned itself before it was twelve months old, owing to the paucity of the secretion. Mrs. W. afterwards had a *slight* return of the catamenia at the regular periods, up to July, 1838, when they entirely ceased. Under these circumstances it was natural to infer that conception could not afterwards take place, but in this conclusion my patient has been deceived, as she was this morning delivered of a healthy boy, her tenth child. Her other children are all living. In this case conception must have taken place at least seven months after what I should have considered to be the final cessation of the menses. JOHN PEARSON.

*Staleybridge*, Dec. 31, 1839.

*Lon. Lan.*

*Chloride of Sodium in Scrofula.*—M. A. Latour speaks highly of the utility of this remedy in scrofula or pulmonary consumption. The following case is illustrative of its effects:

A girl, 13 years of age, of lymphatic temperament, suffered, for more than a year, under scrofulous symptoms; the submaxillary ganglia were greatly enlarged, and the upper lip was the seat of an extensive scrofulous ulceration, for which a variety of remedies had been tried during eleven months without benefit.

On the 9th of April a drachm of sea-salt was given in soup, and ordered to be continued daily. The sore was washed with salt-water, and the diet was confined entirely to animal food. The re-action produced by the salt was so great that the dose was diminished by one-half, and then continued at that dose. The child took frequent exercise in the open air. Towards the middle of May the ulcer was healed, and in fifty days a complete cure was obtained. M. Latour recommends that the salt should be given in flour, made up in the form of a little French roll.

Thus a drachm of salt, dissolved in a small quantity of water, may be mixed with four ounces of flour. Children will readily eat one or two of these rolls in the day.—*Ibid, from L'Experiénce*, Jan. 9, 1840.

---

*Statistical Researches on Pneumonia.*—M. Pelletan, *chef de clinique* of M. Bouillaud, has carefully analysed 75 cases of pneumonia which he observed in the hospital of La Charité during the years 1834, 1835, and 1836. The following deductions may be drawn from these cases:—

Single pneumonia (i. e. affecting one side of the chest only) is more frequent than double pneumonia, in the proportion of 7 to 2. In the 75 cases the right lung was more frequently attacked than the left one, in the proportion of 2½ to 1. The base of the lung was more frequently attacked than the apex, in the proportion of 3 to 2. Men were more frequently attacked than women, in the proportion of 10 to 1. The influence of exposure to cold, as an exciting cause, was found to exist in 7-9th's of the cases. The frequency of the pulse gave no exact indication of the progress of the disease; on the contrary, the frequency of the respiration seemed to measure exactly the degree and danger of the malady.

Prostration of strength and delirium more frequently existed with pneumonia of the apex, than of the base. As for the treatment the greatest benefit was derived from bleeding *coup sur coup.* Only two deaths occurred in 55 cases of simple pneumonia, and the duration of the disease was manifestly abridged. Blisters were of little service in adults; sometimes useful in children, but of very great benefit in old people.—*Ibid, from Gaz. Med.* Jan. 11, 1840.

